# Malignant transformation of uterine leiomyoma to myxoid leiomyosarcoma after morcellation associated with *ALK* rearrangement and loss of 14q

**DOI:** 10.18632/oncotarget.25137

**Published:** 2018-06-12

**Authors:** Carsten Holzmann, Christian Saager, Gunhild Mechtersheimer, Dirk Koczan, Burkhard M. Helmke, Jörn Bullerdiek

**Affiliations:** ^1^ Institute of Medical Genetics, University Rostock Medical Center, Rostock D-18057, Germany; ^2^ Clinic Dr. Hancken, Stade D-21680, Germany; ^3^ Department of General Pathology, Institute of Pathology, University Hospital Heidelberg, Heidelberg D-69120, Germany; ^4^ Institute of Immunology, University Rostock Medical Center, Rostock D-18057, Germany; ^5^ Institute of Pathology, Elbe Clinics, Stade D-21682, Germany; ^6^ Human Genetics, University of Bremen, Bremen D-28359, Germany

**Keywords:** uterine leiomyoma, uterine leiomyosarcoma, morcellation, parasitic leiomyoma, genetic alterations

## Abstract

A 50 year old woman underwent laparoscopic supracervical hysterectomy because of symptomatic fibroids. Histologic examination of samples obtained after morcellation revealed typical uterine leiomyomas in all samples investigated. 28 and 47 months later, respectively, the patient presented with peritoneal spreading of nodules that were surgically removed and histologically classified as leiomyosarcoma. In 3/4 of samples obtained after morcellation copy number/SNP-array hybridization showed complex genomic alterations widely identical to the pattern characterizing the sarcoma. Therefore, we conclude that the leiomyosarcoma had unambiguously developed from one of the leiomyomas as a result of secondary genetic alterations i.e. a rearrangement of *ALK* and a del(14q). The case is challenging the current risk estimates for spreading of unexpected malignant uterine tumors due to power morcellation and highlights the relevance of certain genetic alterations for rare malignant transformation of uterine benign smooth muscle tumors.

## INTRODUCTION

Being part of minimal-invasive removal of uterine leiomyomas or hysterectomy, power morcellation of tissue specimens carries the risk of unexpected spreading of cancerous tissue, e.g. derived from leiomyosarcomas, within the abdomen and pelvis. Thus, the FDA has issued a warning against the use of power morcellation in the majority of women undergoing myomectomy or hysterectomy for treatment of fibroids and to urge the manufacturers of morcellators to include this information in their respective product labels [1].

Unexpected malignant tumors are a rare finding after surgery for symptomatic leiomyomas but there is little doubt that morcellation of these lesions is associated with a higher risk of iatrogenic peritoneal spread compared to women having surgery without morcellation [2]. Accordingly, an appropriate pre-procedure briefing about the pros and cons of minimal-invasive surgery with morcellation based on realistic risk figures on the unexpected spreading of a “dangerous” tumor is recommended. Currently, risk figures are mostly based on retrospective analyses of the tissue removed and vary over a broad range [3] On the other hand, follow-up of those tumors classified as benign is usually lacking [4–6] though the results of thorough histologic examinations as well as of recent genetic analyses suggest that, albeit rarely, leiomyomas can undergo malignant transformation and that a considerable percentage of leiomyosarcomas still contain areas displaying “benign” histology [7]. *Vice versa*, leiomyomas may contain small areas with malignant transformation that escape initial diagnosis but later can give rise to local recurrences and metastases. It seems tempting to assume that data obtained after tumor morcellation [8–11] are not suited to correctly assess the risk to benefit ratio of morcellation merely resulting from statistical problems [12]: A large percentage of patients have more than one tumor but once the specimen has been morcellated it is, as a rule, no longer possible to allocate the sample to an individual tumor despite some sampling recommendations [13].

Here, a case is presented that showed a benign histology at initial examination. The samples obtained from initial surgery as well as from a malignant tumor surgically removed two years later shared a pattern of characteristic genetic abnormalities unambiguously demonstrating a common clonal origin. Nevertheless, few of these abnormalities allowed to distinguish between the benign tumor and its malignant counterpart. Though being a single case only, it clearly underlines the necessity of prospective studies for risk assessment of power morcellation.

## RESULTS

**Figure 1:**
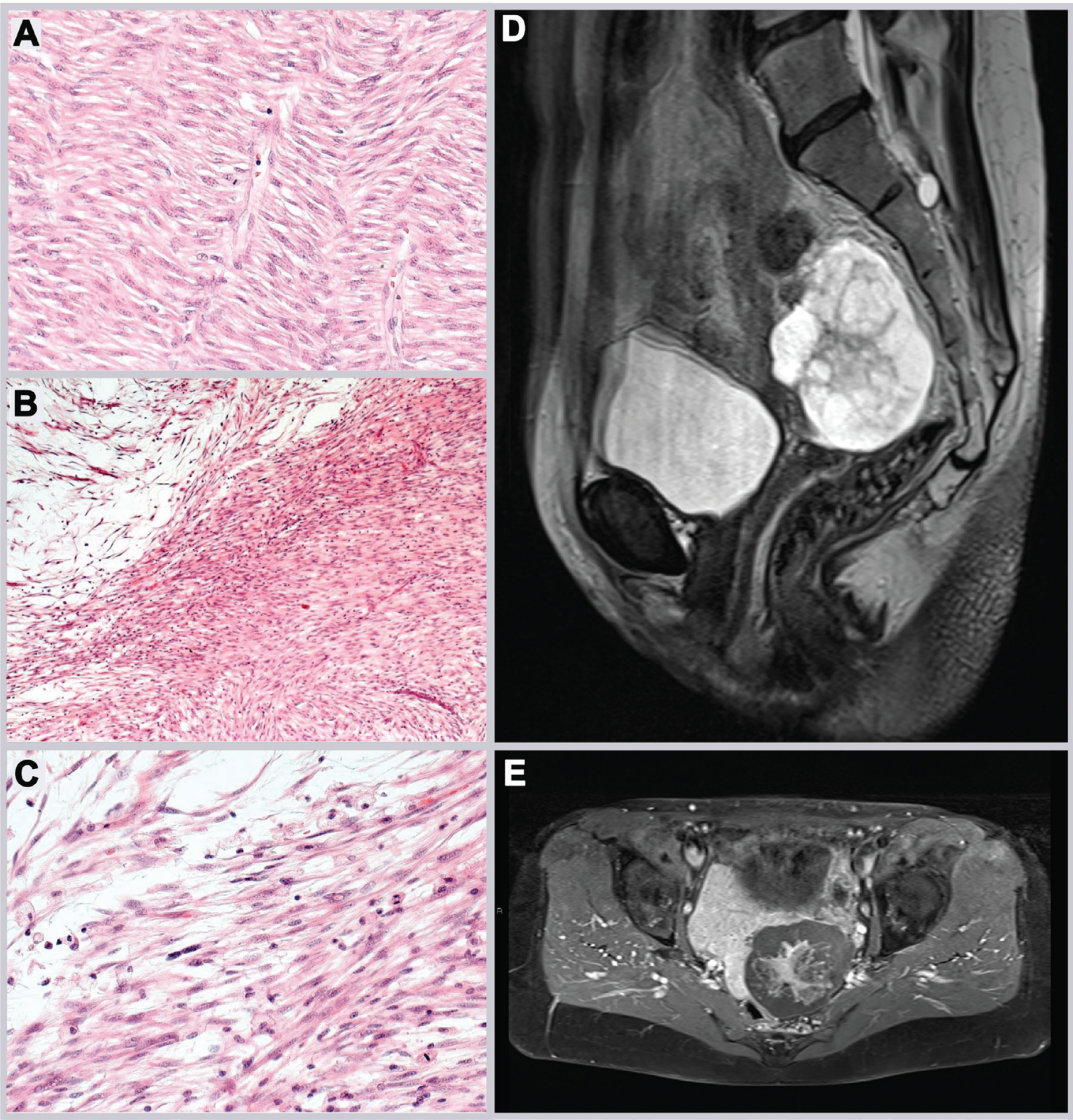
Histology and MRI of tumors studied by CNV and gene expression arrays, respectively (**A**) Morcellated specimens of leiomyoma removed by initial surgery histologically showing fascicles of isomorphic spindled cells with cigar-shaped nuclei, indistinct borders, and eosinophilic cytoplasm (cf. Figure 2C). (**B**, **C**) Leiomyosarcoma with hypocellular myxoid areas and atypical spindle cells with mitotic activity (cf. Figure 2A). (**D**) Sagittal MRI T2 imaging shows a well defined presacral mass of up to 6 cm diameter in axial orientation with inhomogenous signal intensity, apparently mainly cystic with solid central parts. Adjacent structures do not show signs of infiltration. (**E**) MRI T1 imaging post i.v. contrast application shows enhancement of the central solid parts and a cleary defined border towards rectum and bladder.

A fifty-year-old patient with intervertebral disc degeneration and multiple uterine fibroids decided to have laparoscopic supracervical hysterectomy (LASH). When she was admitted to the hospital ultrasound showed a 16-week size uterus with normal ovaries. After LASH, five tumor samples were paraffin-embedded for histologic examination which in all samples confirmed the presence of leiomyomas (Figure 1A). In none of the samples evidence for a smooth muscle tumor of uncertain malignant potential (STUMP) or a leiomyosarcoma, respectively, was found.

**Figure 2:**
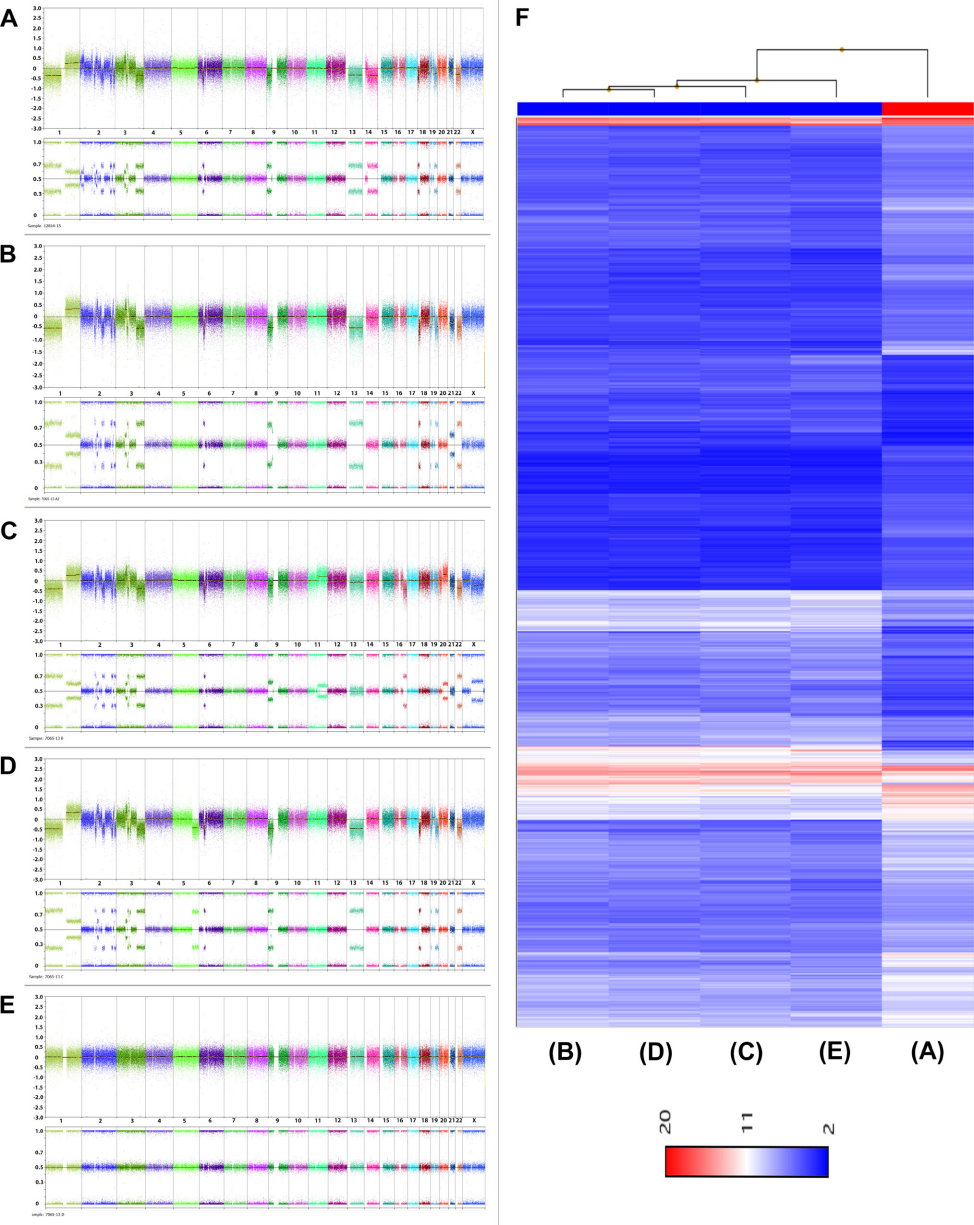
Genomic overview showing the results of CNV arrays (**A**–**E**) and hierarchical clustering displaying gene expression (**F**). (A) and F, 5th lane: leiomyosarcoma, (B–E and F), 1st to 4th lane: leiomyoma samples obtained during initial surgery. Top panel in (A–E) gives the copy number probe intensity calls and the bottom panel displays the calculation of B-allele frequencies (BAF).

**Figure 3:**
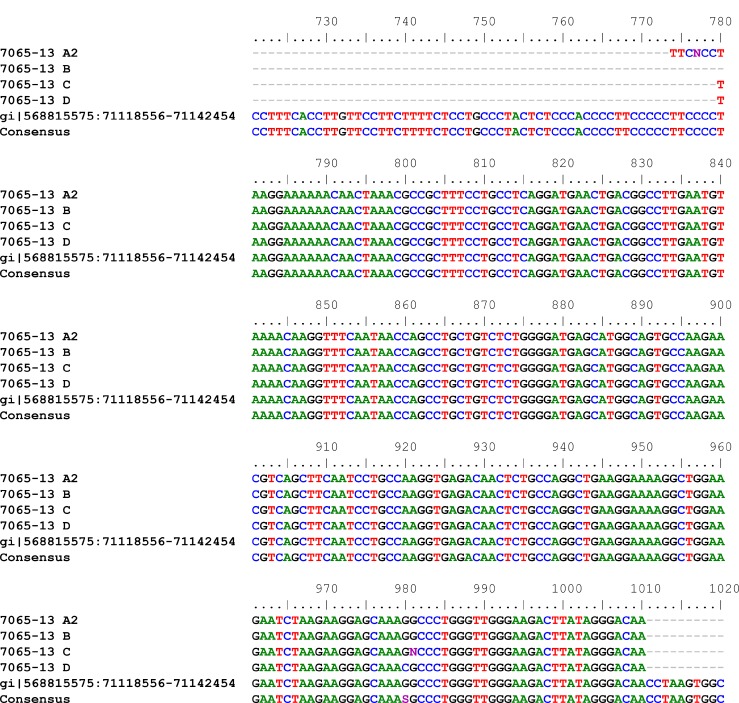
Results of *MED12* mutation analyses of four samples obtained after morcellation and classified as leiomyoma

28 months later the patient had sonography during routine follow-up examination showing a cystic pelvic mass measuring 7.8 × 5.1 × 7.1 cm which was removed by laparotomy and histologically classified as myxoid leiomyosarcoma (Figure 1B–1E). Because of a possible origin of the leiomyomsarcoma from a morcellated tumor, genomic profiles from four samples obtained from initial surgery as well as from the tumor that was detected 28 month later were investigated by copy number/SNP-array hybridization using the molecular inversion probe (MIP) technology based copy number/SNP-array hybridization using the OncoScan platform (Affymetrix, described previously [14]) in combination with expression profiling applying the WT Pico protocol on Affymetrix ClariomD™ arrays. Four samples (Figure 2A–2E) displayed gross genomic alterations with a striking non-random similarity noted between three of the initial samples (Figure 2B–2E) and the leiomysarcoma (Figure 2A) (for details see Supplementary Table 1). In contrast, one of the samples did not show any apparent genomic gains or losses, respectively, suggesting its origin from an independent leiomyoma. Gene expression analysis carried out from the same samples used for MIP array hybridization revealed a strong transcriptomic similarity between the three UL samples with almost identical genomic profiles but not with the leiomyosarcoma despite their corresponding genomic profile (Figure 2F). Overexpression of *HMGA2* indicating a rearrangement of this gene by chromosomal translocation was detected in none of the samples (as compared to tumors with cytogenetically detectable rearrangements of the 12q14-15 region, data not shown). Also, in none of the samples obtained after morcellation, *MED12* mutations of exon 2 or of the intron 1/exon 2 boundary constituting characteristic mutations in a large subgroup of fibroids were seen (Figure 3).

**Figure 4:**
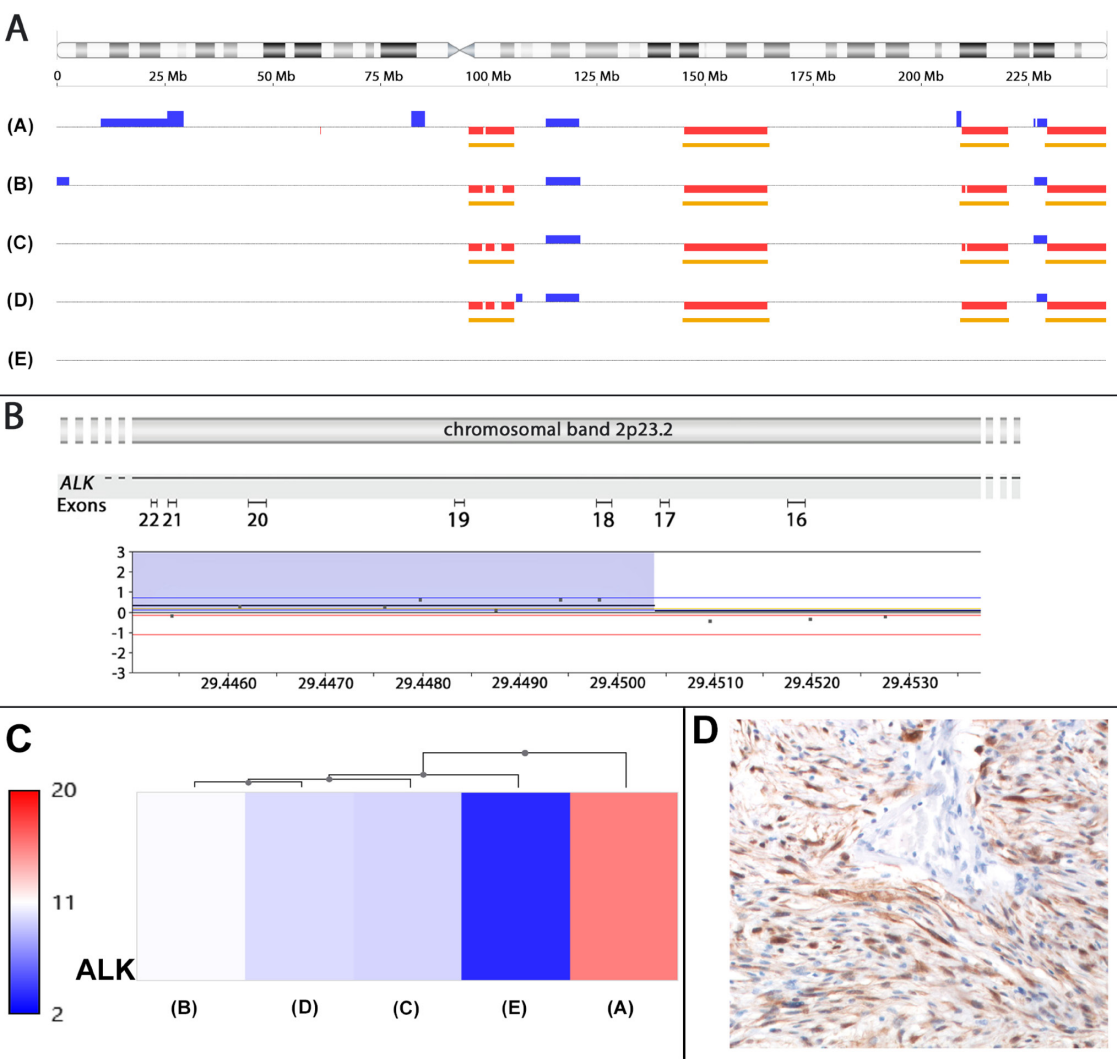
*ALK* amplification and rearrangement in the leiomyosarcoma (**A**) Ideogram of chromosome 2 (top) and gains and losses affecting this chromosome in the leiomyosarcoma (A) as well as in 3/4 (B–D) samples obtained during initial surgery histologically showing a leiomyoma. In the remaining sample (E) no gains or losses were observed indicating a different clonally unrelated leiomyoma. (**B**) High resolution of part of a gain involving chromosomal subband 2p23.2 with the corresponding intron-exon structure of *ALK*. (**C**) overexpression of *ALK* mRNA (A). The similarity of expression is indicated by the dendogram on top of the illustration. (**D**) Strong expression of ALK as shown immunohistochemically.

Next, an attempt was made to define those genetic alterations distinguishing the malignant tumor from its still benign counterpart. As a result, the following genetic alterations were found to be confined to the sarcoma sample: The sarcoma showed an apparently terminal loss of a large approximately 69.66 Mb segment of the long arm of chromosome 14 (14q13.3-14qter, positions 36,876,044–106,531,400). Furthermore, only the sarcoma sample showed an additional small gain on the long arm of chromosome 2 (2q33.3-2q34) and also changes resembling “healed” chromothripsis also of the short arm of chromosome 2 leading to two gained segments (Figure 4A). Interestingly, these alterations indicate a rearranged and apparently amplified allele of the *ALK* (anaplastic lymphoma receptor tyrosine kinase) gene assigned to chromosomal band 2p23 with an intragenic break that could be narrowed down to a region between intron 17 and intron 19 (Figure 4B). Because of its relevance for targeted therapies we were interested to see if this rearrangement and the amplification did also affect the expression level of *ALK*. *ALK* mRNA expression in the malignant sample was clearly elevated when compared with the other four samples using expression arrays (Figure 4C). Immunohistochemical staining for ALK was performed using the corresponding antibody (Zeta Corporation, Sierra Madre, CA, USA, clone 1A4), a detection kit (DAKO ChemMate; DAKO, Glostrup, Denmark) and a semiautomated stainer (DAKO; TechMate) according to the specifications of the manufacturer. For antigen retrieval, the slides were treated in a PT Link module (DAKO) using the EnVision™ FLEX Target Retrieval Solution, low pH (DAKO). The antibody dilution used was 1:50. For negative control the primary antibody was omitted. By immunostaining strong diffuse expression of the protein was noted in the malignant sample (Figure 4D). In contrast, all samples obtained after morcellation lacked ALK positivity.

19 months after this latter surgery, the patient was again admitted to the hospital because of abdominal pain. Magnetic resonance imaging (MRI) revealed a 10 × 8 × 6 cm abdominal mass attached to the liver and numerous other nodules attached to the abdominal wall that were surgically removed and histologically classified as myxoid leiomyosarcoma as well.

## DISCUSSION

Counselling about different alternatives of fibroid removal or hysterectomy, respectively, should include appropriate risk estimates for direct risks associated with the procedure, i.e. open versus minimal-invasive surgery, as well as for spreading of unexpected malignant tumors [15]. Nevertheless, the risk figures reported for spreading of unexpected malignancies due to tissue morcellation during hysterectomy or myomectomy strongly vary. As to possible reasons for this varying rigor several explanations have been suggested including risk factors, such as age that are poorly stratified as yet, and diagnostic criteria for leiomyosarcoma differing between studies from different periods of time [15] Assuming base-case estimates for procedure-related deaths Siedhoff *et al.* [16] have concluded that at a hypothetical leiomyosarcoma incidence of 0.0015 an equivalent mortality between both groups, i.e. laparoscopic *versus* abdominal hysterectomy, is given. As to their sources regarding the incidence of leiomyosarcoma among women undergoing hysterectomy due to presumed fibroids, an estimate of 0.0012 was obtained leading to the conclusion that laparoscopic hysterectomy resulted in more quality-adjusted life years than abdominal hysterectomy (499.171 *vs*. 490.711 over five years).

We feel that the present case is challenging previous risk estimates. Described is a patient where initial examination did not provide any evidence for the existence of a malignant smooth muscle tumor. Healed chromothripsis affecting chromosome 2 and chromosome 3 had resulted in a highly characteristic and unique finger-print like pattern of gains and losses of chromosomal segments. This was found not only in some of the samples resulting from morcellation but also in a leiomyosarcoma first detected more than two years later. Thus, the leiomyosarcoma is likely to have originated from a pre-existing leiomyoma. Its myxoid differentiation associated with a rearranged and apparently amplified allele of *ALK* fits with these genetic alterations seen in smooth muscle tumors in general [17] as well as in uterine myelofibroblastic tumors displaying myxoid features [18, 19] and may offer novel options for targeted therapy [20].

Previous investigations clearly point to a number of different genetic subtypes of uterine leiomyomas with those affected by mutations of *MED12* representing by far the most frequent sub-entity followed by that showing rearrangements of *HMGA2* [14, 21]. Apparently both types of mutually exclusive mutations have been excluded in the present tumor undergoing malignant transformation. Instead, losses of 22 in all samples of the same tumor and losses of 19 in the majority of the samples fit with the results of Christacos *et al.* [22] demonstrating that loss of 1p was often associated with losses of chromosomes 19 and/or 22 and is constituting an own albeit rare genetic subgroup of uterine leiomyomas. In line with these findings the tumor described here as well as its recurrence had a deletion of 1p. Nevertheless, there is no evidence supporting a classification of the primary tumor different from leiomyoma.

**Figure 5:**
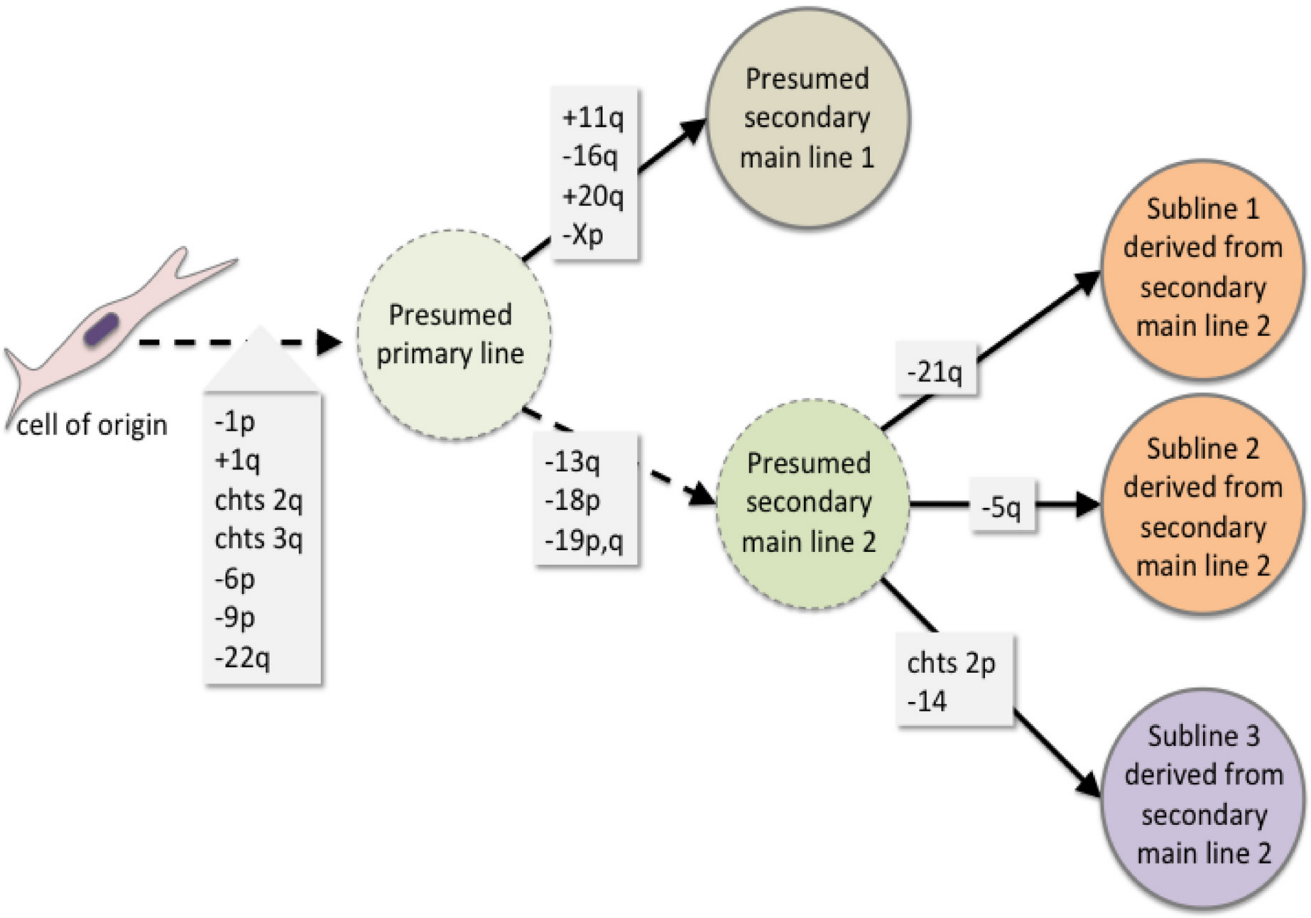
Scheme summarizing the proposed genetic evolution of the tumor cell population leading to intratumoral genetic heterogeneity and malignant transformation, respectively

Accordingly, this case would have been missed by nearly all of the studies underlying current risk estimates for the occurrence of unexpected leiomyosarcoma since at the time of initial surgery no evidence for malignancy was obtained. As to the molecular pathogenesis it is likely to asssume that one of the fibroids was characterized by genetic heterogeneity due to extended karyotype evolution (Figure 5). Morcellation had resulted in spreading and ectopically re-seeding of a population of cells which were present and had undergone malignant transformation either already at the time of morcellation or first gave rise to a so-called parasitic leiomyoma with subsequent malignant transformation. Based on a recent review of the literature the overall incidence of parasitic myomas after laparoscopic morcellation is in the range 0.12–0.95% [20] but there are as yet no sufficient data giving an impression if and how often parasitic leiomyomas can transform to STUMPs or even leiomyosarcomas. If it turns out that parasitic leiomyomas are at increased risk to transform to leiomyosarcoma previous data for the risk associated with power morcellation have to be revisited. Likewise, prospective studies with a sufficient follow-up of all patients including those with an apparently benign disease for a period of some years seem to be necessary to gain sufficient data to be used for therapeutic decisions.

## MATERIALS AND METHODS

### Tumor samples

Immediately after surgery tumor samples were fixed in paraformaldehyde (4% in PBS), and processed for paraffin embedding.

For histological examination representative samples of the tumors were fixed in paraformaldehyde (4% in PBS), and processed for paraffin embedding according to standard techniques. For histological examination tissue sections from paraffin embedded (FFPE) samples (1–2 μm) were de-paraffinized in xylene, rehydrated through a series of ethanol, and stained with hematoxylin/eosin.

### Immunohistochemical staining (IHC)

IHC for ALK was performed using the corresponding antibody (Zeta Corporation, Sierra Madre, CA, USA, clone 1A4), a detection kit (DAKO ChemMate; DAKO, Glostrup, Denmark) and a semiautomated stainer (DAKO; TechMate) according to the specifications of the manufacturer. For antigen retrieval, the slides were treated in a PT Link module (DAKO) using the EnVision™ FLEX Target Retrieval Solution, low pH (DAKO). The antibody dilution used was 1:50. For negative control the primary antibody was omitted.

### Analyses for mutations of *MED12*

Analyses for mutations of *MED12* were performed as described previously [14, 21]. For *MED12* sequencing the following primers previously described by Mäkinen et al. [23] were used:

*MED12* FP: gccctttcaccttgttcctt

*MED12* RP: tgtccctataagtcttcccaacc

### Simultaneous extraction and purification of DNA and RNA from FFPE tissue samples using Covaris Adaptive Focused Acoutics (AFA™) and truXTRAC™ FFPE DNA and RNA kits and array hybridization

For array hybridization DNA and RNA extraction (truXTRAC™, Covaris) was done simultaneously from one single 10 μm FFPE section each. The emulsification of the paraffin was done applying 5 minutes focused acoustic waves of 75 watts to a 10 µm FFPE section using a Covaris M220 device (Woburn, Massachusetts, USA). Proteinase K digestion was performed at 56° C for 15 minutes. The paraffin including the genomic DNA was sedimented at 16.000 g. For RNA isolation the supernatant was heated to 80° C to reverse the formaldehyde crosslink followed by a DNaseI digestion step and a spincolumn cleanup procedure. Accordingly the pellet was subjected to spincolumn cleanup of the genomic DNA after an additional proteinase K digestion. Fluorometric quantification was performed with the Qubit™ 2.0 fluorometer using the Qubit™ dsDNA HS- and RNA HS Assay Kit (Thermo Fisher Scientific).

The samples were then subjected to MIP-based copy number/SNP-array hybridization using the OncoScan platform (Affymetrix, described previously [14]) in combination with expression profiling applying the WT Pico protocol on Affymetrix ClariomD^™^ arrays. To avoid an over amplication we were using 45 ng degraded whole RNA, which is close to the maximum recommended by the supplier (0.5 to 50 ng). First strand cDNA synthesis is introducing by N6- and Oligo-dT priming a T7 promoter sequence 5extended with an universal PCR primer site. To perform a preamplification (Pre-IVT Amplification) the primer site at the 5`ends of the first cDNA strand is countered by a second strand synthesis random primed (N6) with an adaptor attaching the reverse primer site to each end of the second strand fragments produced by Klenow polymerase. These templates were amplified exponentially for 6 cycles followed by a linear amplification step using the T7 promoter in an over night reaction (IVT, 14 h). Using 20 µg of each aRNA sample after purification as template in a reverse transcription reaction a strand-identical single strand DNA was produced by adding random primers and dNTPs. To enable an endpoint fragmentation reaction a certain fraction of dTTP is replaced by dUTP. After complete RNA removal (RNaseH) the enzymatic fragmentation is performed by uracil deglycosidase for removing uracil in combination with apurinic apyrimidinic endonuclease 1, which is breaking the free endonucleolytic phosphodiester bonds. Desoxynucleotidyl-transferase is adding Biotin-11-dXTP to the 3ends. The hybridization was carried out at 45° C in the GeneChipR Hybridization Oven 645 (Affymetrix) over night. Washing and staining was done with the GeneChipR Fluidic Station 450 according to the suppliers instructions. The prepared microarrays were scanned using the GeneChipR Scanner 3000 at 0.7 micron resolution. Probe level analysis with SST-RMA (signal space transformation-robust multichip average) and data visualization was done with TAC (Transcriptome Analysis Console 4.0; Applied Biosystems).

All data obtained by array analyses have been deposited at the GEO repository (GEO record GSE103050).

## SUPPLEMENTARY MATERIALS TABLE




