# Supplementary material for: Malignant transformation of uterine leiomyoma to myxoid leiomyosarcoma after morcellation associated with ALK rearrangement and loss of 14q
Source: Oncotarget. 2018 Jun 12;9(45):27595–604. doi: 10.18632/oncotarget.25137 (PMC6021249; doi:10.18632/oncotarget.25137)
Supplement: Supplementary file 1 [file oncotarget-09-27595-s001.pdf]

Malignant transformation of uterine leiomyoma to myxoid leiomyosarcoma after morcellation associated with ALK rearrangement and loss of 14q

SUPPLEMENTARY MATERIALS

Supplementary Table 1: Genomic array results. See Supplementary_Table_1
