# Supplementary material for: Malignant transformation of uterine leiomyoma to myxoid leiomyosarcoma after morcellation associated with ALK rearrangement and loss of 14q
Source: Oncotarget. 2018 Jun 12;9(45):27595–604. doi: 10.18632/oncotarget.25137 (PMC6021249; doi:10.18632/oncotarget.25137)
Supplement: Supplementary file 2 [file oncotarget-09-27595-s002.docx]

**Supplemental Table 1**Genomic array results. OncoScan FFPE Assay (Affymetrix, Santa Clara, CA) based on Molecular Inversion Probe (MIP) technology was performed following the manufacturer’s instructions. Arrays were analyzed using the Nexus Copy number 9 Software (BioDiscovery, El Segundo, CA, USA). For the purpose of improved clarity only gains and losses ≥200kb and LOHs ≥5000 kb are shown. Aberrations exclusively found in the leiomyosarcoma are marked in red. For probe number assignment to Fig. 2:

sample 12814-15: Fig. 2A

sample 7065-13 A2: Fig. 2B

sample 7065-13 B: Fig. 2C

sample 7065-13 C: Fig. 2D

sample 7065-13 D: Fig. 2E

| Sample | Chromosome Region | Event | Length | Band | % of CNV Overlap | Probe Median | Probes |
| --- | --- | --- | --- | --- | --- | --- | --- |
| 7065-13 C | chr1:110,269,001-121,350,934 | CN Loss | 11081934 | p13.3 - p11.2 | 36.22 | -0.51 | 898 |
| 7065-13 B | chr1:145,205,788-190,410,612 | CN Gain | 45204825 | q21.1 - q31.1 | 31.02 | 0.26 | 3558 |
| 7065-13 A2 | chr1:145,226,808-190,410,612 | CN Gain | 45183805 | q21.1 - q31.1 | 30.99 | 0.31 | 3554 |
| 7065-13 C | chr1:145,394,955-190,410,612 | CN Gain | 45015658 | q21.1 - q31.1 | 30.73 | 0.31 | 3539 |
| 12814-15 | chr1:146,571,244-182,508,761 | CN Gain | 35937518 | q21.1 - q25.3 | 28.86 | 0.23 | 2871 |
| 12814-15 | chr1:183,134,854-212,314,575 | CN Gain | 29179722 | q25.3 - q32.3 | 19.52 | 0.25 | 2160 |
| 7065-13 B | chr1:190,443,316-190,683,392 | CN Loss | 240077 | q31.1 | 13.75 | -0.22 | 18 |
| 7065-13 B | chr1:190,695,904-212,314,575 | CN Gain | 21618672 | q31.1 - q32.3 | 15.20 | 0.27 | 1561 |
| 7065-13 C | chr1:190,695,904-212,314,575 | CN Gain | 21618672 | q31.1 - q32.3 | 15.20 | 0.32 | 1561 |
| 7065-13 A2 | chr1:190,709,144-212,314,575 | CN Gain | 21605432 | q31.1 - q32.3 | 15.15 | 0.33 | 1560 |
| 12814-15 | chr1:213,006,397-249,212,878 | CN Gain | 36206482 | q32.3 - q44 | 28.35 | 0.27 | 2856 |
| 7065-13 A2 | chr1:213,037,310-249,212,878 | CN Gain | 36175569 | q32.3 - q44 | 28.34 | 0.33 | 2854 |
| 7065-13 B | chr1:213,037,310-249,212,878 | CN Gain | 36175569 | q32.3 - q44 | 28.34 | 0.29 | 2854 |
| 7065-13 C | chr1:213,037,310-249,212,878 | CN Gain | 36175569 | q32.3 - q44 | 28.34 | 0.33 | 2854 |
| 7065-13 C | chr1:754,192-110,221,639 | CN Loss | 109467448 | p36.33 - p13.3 | 20.52 | -0.49 | 8815 |
| 12814-15 | chr1:754,192-121,350,934 | CN Loss | 120596743 | p36.33 - p11.2 | 21.99 | -0.38 | 9731 |
| 12814-15 | chr1:754,192-121,350,934 | LOH | 120596743 | p36.33 - p11.2 | 21.99 | -0.38 | 9731 |
| 7065-13 A2 | chr1:754,192-121,350,934 | CN Loss | 120596743 | p36.33 - p11.2 | 21.99 | -0.48 | 9731 |
| 7065-13 A2 | chr1:754,192-121,350,934 | LOH | 120596743 | p36.33 - p11.2 | 21.99 | -0.48 | 9731 |
| 7065-13 B | chr1:754,192-121,350,934 | CN Loss | 120596743 | p36.33 - p11.2 | 21.99 | -0.42 | 9731 |
| 7065-13 B | chr1:754,192-121,350,934 | LOH | 120596743 | p36.33 - p11.2 | 21.99 | -0.42 | 9731 |
| 7065-13 C | chr1:754,192-121,350,934 | LOH | 120596743 | p36.33 - p11.2 | 21.99 | -0.50 | 9731 |
| 12814-15 | chr10:45,672,429-45,924,023 | CN Loss | 251595 | q11.21 | 59.05 | -0.28 | 18 |
| 7065-13 A2 | chr10:75,993,245-76,263,261 | CN Loss | 270017 | q22.2 | 100.00 | -0.29 | 18 |
| 7065-13 A2 | chr10:92,162,285-92,470,777 | CN Gain | 308493 | q23.31 | 100.00 | 0.16 | 22 |
| 7065-13 B | chr11:119,142,244-120,018,743 | High Copy Gain | 876500 | q23.3 | 5.09 | 0.33 | 72 |
| 7065-13 B | chr11:120,037,862-134,938,847 | CN Gain | 14900986 | q23.3 - q25 | 22.25 | 0.18 | 1184 |
| 7065-13 A2 | chr11:55,339,652-55,584,109 | CN Loss | 244458 | q11 | 100.00 | -0.26 | 18 |
| 7065-13 B | chr11:65,676,516-71,339,667 | CN Gain | 5663152 | q13.1 - q13.4 | 19.28 | 0.24 | 547 |
| 7065-13 B | chr11:71,739,423-76,214,009 | CN Gain | 4474587 | q13.4 - q13.5 | 27.08 | 0.16 | 326 |
| 12814-15 | chr11:75,583,990-75,941,236 | CN Loss | 357247 | q13.5 | 89.61 | -0.19 | 25 |
| 7065-13 B | chr11:76,418,138-88,489,493 | CN Gain | 12071356 | q13.5 - q14.3 | 19.85 | 0.22 | 864 |
| 7065-13 B | chr11:88,504,534-89,880,469 | CN Gain | 1375936 | q14.3 | 100.00 | 0.21 | 129 |
| 7065-13 B | chr11:89,895,940-119,140,832 | CN Gain | 29244893 | q14.3 - q23.3 | 23.09 | 0.19 | 2299 |
| 7065-13 B | chr12:28,172,146-28,810,322 | CN Loss | 638177 | p11.22 | 32.08 | -0.20 | 43 |
| 7065-13 C | chr12:32,250,251-32,668,892 | CN Loss | 418642 | p11.21 | 92.81 | -0.58 | 31 |
| 12814-15 | chr13:19,084,823-115,103,150 | CN Loss | 96018328 | q11 - q34 | 22.07 | -0.35 | 7934 |
| 12814-15 | chr13:19,084,823-115,103,150 | LOH | 96018328 | q11 - q34 | 22.07 | -0.35 | 7934 |
| 7065-13 A2 | chr13:19,084,823-115,103,150 | CN Loss | 96018328 | q11 - q34 | 22.07 | -0.47 | 7934 |
| 7065-13 A2 | chr13:19,084,823-115,103,150 | LOH | 96018328 | q11 - q34 | 22.07 | -0.47 | 7934 |
| 7065-13 C | chr13:19,084,823-115,103,150 | CN Loss | 96018328 | q11 - q34 | 22.07 | -0.49 | 7934 |
| 7065-13 C | chr13:19,084,823-115,103,150 | LOH | 96018328 | q11 - q34 | 22.07 | -0.49 | 7934 |
| 7065-13 B | chr13:19,084,823-32,852,184 | CN Loss | 13767362 | q11 - q13.1 | 40.34 | -0.09 | 1074 |
| 7065-13 B | chr13:34,620,493-42,285,381 | CN Loss | 7664889 | q13.2 - q14.11 | 14.00 | -0.10 | 633 |
| 7065-13 B | chr13:42,299,822-42,551,508 | CN Loss | 251687 | q14.11 | 62.65 | -0.22 | 18 |
| 7065-13 B | chr13:42,570,201-45,384,736 | CN Loss | 2814536 | q14.11 - q14.12 | 16.87 | -0.13 | 189 |
| 7065-13 B | chr13:45,408,789-45,662,375 | CN Loss | 253587 | q14.12 | 1.09 | -0.22 | 18 |
| 7065-13 B | chr13:45,989,456-48,969,515 | CN Loss | 2980060 | q14.13 - q14.2 | 38.81 | -0.14 | 351 |
| 7065-13 B | chr13:49,015,533-63,593,263 | CN Loss | 14577731 | q14.2 - q21.31 | 16.53 | -0.09 | 1018 |
| 7065-13 B | chr13:64,210,125-64,626,247 | CN Loss | 416123 | q21.31 | 94.10 | -0.20 | 29 |
| 7065-13 B | chr13:65,583,733-65,983,547 | CN Loss | 399815 | q21.31 - q21.32 | 0.68 | -0.24 | 27 |
| 7065-13 B | chr13:67,852,176-68,189,484 | CN Loss | 337309 | q21.32 | 48.85 | -0.29 | 23 |
| 7065-13 B | chr13:79,081,790-79,363,397 | CN Loss | 281608 | q31.1 | 4.23 | -0.19 | 21 |
| 7065-13 B | chr13:84,604,038-93,382,618 | CN Loss | 8778581 | q31.1 - q31.3 | 19.53 | -0.08 | 751 |
| 7065-13 B | chr13:93,396,851-93,755,828 | CN Loss | 358978 | q31.3 | 9.45 | -0.19 | 37 |
| 7065-13 B | chr13:96,602,734-113,094,395 | CN Loss | 16491662 | q32.1 - q34 | 15.05 | -0.10 | 1730 |
| 7065-13 D | chr14:106,537,284-106,777,972 | CN Loss | 240689 | q32.33 | 100.00 | -0.96 | 47 |
| 12814-15 | chr14:106,537,284-106,779,252 | Homozygous Copy Loss | 241969 | q32.33 | 100.00 | -1.88 | 48 |
| 7065-13 A2 | chr14:106,537,284-106,779,252 | Homozygous Copy Loss | 241969 | q32.33 | 100.00 | -1.01 | 48 |
| 7065-13 B | chr14:106,537,284-106,789,744 | Homozygous Copy Loss | 252461 | q32.33 | 100.00 | -0.90 | 49 |
| 7065-13 C | chr14:106,537,284-106,789,744 | Homozygous Copy Loss | 252461 | q32.33 | 100.00 | -0.86 | 49 |
| 12814-15 | chr14:106,789,744-107,282,024 | CN Loss | 492281 | q32.33 | 100.00 | -0.33 | 91 |
| 12814-15 | chr14:20,219,083-20,571,795 | CN Gain | 352713 | q11.2 | 100.00 | 0.17 | 19 |
| 12814-15 | chr14:22,611,697-26,877,020 | CN Loss | 4265324 | q11.2 - q12 | 30.79 | -0.11 | 332 |
| 12814-15 | chr14:36,582,940-107,282,024 | LOH | 70699085 | q13.2 - q32.33 | 25.17 | -0.38 | 5450 |
| 12814-15 | chr14:36,876,044-106,531,400 | CN Loss | 69655357 | q13.3 - q32.33 | 24.40 | -0.38 | 5289 |
| 7065-13 B | chr14:56,271,006-56,551,152 | CN Loss | 280147 | q22.3 | 0.68 | -0.22 | 19 |
| 7065-13 B | chr14:64,474,121-64,710,417 | CN Loss | 236297 | q23.2 | 100.00 | -0.18 | 31 |
| 7065-13 B | chr14:83,696,310-83,950,186 | CN Loss | 253877 | q31.2 | 18.31 | -0.23 | 18 |
| 12814-15 | chr15:31,063,058-31,307,753 | CN Loss | 244696 | q13.2 - q13.3 | 97.61 | -0.18 | 28 |
| 12814-15 | chr15:31,324,531-31,633,161 | CN Gain | 308631 | q13.3 | 53.46 | 0.15 | 22 |
| 7065-13 D | chr15:76,917,885-77,910,095 | CN Loss | 992211 | q24.3 | 100.00 | -0.20 | 66 |
| 7065-13 B | chr15:93,289,466-93,564,972 | CN Loss | 275507 | q26.1 | 0.67 | -0.32 | 19 |
| 12814-15 | chr16:1,599,913-2,063,481 | CN Gain | 463569 | p13.3 | 38.91 | 0.18 | 31 |
| 7065-13 A2 | chr16:1,720,844-2,003,123 | CN Gain | 282280 | p13.3 | 43.17 | 0.25 | 19 |
| 7065-13 B | chr16:1,720,844-2,003,123 | CN Gain | 282280 | p13.3 | 43.17 | 0.19 | 19 |
| 12814-15 | chr16:31,933,352-33,619,415 | CN Gain | 1686064 | p11.2 | 100.00 | 0.19 | 34 |
| 7065-13 D | chr16:31,933,352-33,619,415 | CN Gain | 1686064 | p11.2 | 100.00 | 0.24 | 34 |
| 7065-13 A2 | chr16:34,579,231-34,874,339 | CN Loss | 295109 | p11.2 - p11.1 | 100.00 | -0.23 | 20 |
| 12814-15 | chr16:4,838,617-5,182,520 | CN Gain | 343904 | p13.3 | 56.18 | 0.20 | 23 |
| 7065-13 B | chr16:57,046,687-57,303,156 | CN Gain | 256470 | q13 | 0.79 | 0.16 | 18 |
| 7065-13 B | chr16:65,011,855-90,158,005 | LOH | 25146151 | q21 - q24.3 | 27.26 | -0.43 | 2062 |
| 7065-13 B | chr16:65,165,092-90,158,005 | CN Loss | 24992914 | q21 - q24.3 | 27.43 | -0.43 | 2032 |
| 12814-15 | chr16:75,230,230-75,448,921 | CN Loss | 218692 | q23.1 | 36.13 | -0.32 | 27 |
| 12814-15 | chr16:88,953,221-89,199,651 | CN Loss | 246431 | q24.3 | 100.00 | -0.36 | 27 |
| 7065-13 A2 | chr17:1,018,393-1,358,205 | CN Gain | 339813 | p13.3 | 13.37 | 0.22 | 46 |
| 12814-15 | chr17:36,242,848-36,868,772 | CN Gain | 625925 | q12 | 82.30 | 0.18 | 44 |
| 12814-15 | chr17:39,811,683-40,094,835 | CN Gain | 283153 | q21.2 | 36.18 | 0.16 | 20 |
| 7065-13 C | chr17:42,721,039-42,974,143 | CN Gain | 253105 | q21.31 | 4.47 | 0.18 | 18 |
| 7065-13 B | chr17:44,958,022-45,298,408 | CN Loss | 340387 | q21.32 | 93.81 | -0.18 | 23 |
| 12814-15 | chr17:58,380,096-58,696,365 | CN Gain | 316270 | q23.2 | 33.14 | 0.15 | 26 |
| 12814-15 | chr18:12,842-15,377,471 | CN Loss | 15364630 | p11.32 - p11.21 | 31.07 | -0.37 | 1072 |
| 12814-15 | chr18:12,842-15,377,471 | LOH | 15364630 | p11.32 - p11.21 | 31.07 | -0.37 | 1072 |
| 7065-13 A2 | chr18:12,842-15,377,471 | CN Loss | 15364630 | p11.32 - p11.21 | 31.07 | -0.47 | 1072 |
| 7065-13 A2 | chr18:12,842-15,377,471 | LOH | 15364630 | p11.32 - p11.21 | 31.07 | -0.47 | 1072 |
| 7065-13 C | chr18:12,842-15,377,471 | CN Loss | 15364630 | p11.32 - p11.21 | 31.07 | -0.49 | 1072 |
| 7065-13 C | chr18:12,842-15,377,471 | LOH | 15364630 | p11.32 - p11.21 | 31.07 | -0.49 | 1072 |
| 12814-15 | chr18:45,274,390-45,525,566 | CN Loss | 251177 | q21.1 | 17.27 | -0.29 | 19 |
| 7065-13 B | chr18:62,367,627-62,661,788 | CN Gain | 294162 | q22.1 | 1.73 | 0.17 | 19 |
| 12814-15 | chr19:10,422,004-11,770,579 | CN Gain | 1348576 | p13.2 | 9.05 | 0.22 | 120 |
| 12814-15 | chr19:11,796,869-12,989,560 | CN Loss | 1192692 | p13.2 | 47.93 | -0.29 | 94 |
| 7065-13 C | chr19:11,819,193-12,480,037 | CN Loss | 660845 | p13.2 | 49.21 | -0.52 | 45 |
| 7065-13 A2 | chr19:11,819,193-12,989,560 | CN Loss | 1170368 | p13.2 | 48.85 | -0.43 | 92 |
| 7065-13 C | chr19:12,493,192-13,213,967 | CN Loss | 720776 | p13.2 | 34.56 | -0.21 | 68 |
| 12814-15 | chr19:14,093,598-18,251,438 | CN Gain | 4157841 | p13.12 - p13.11 | 20.64 | 0.12 | 353 |
| 7065-13 C | chr19:35,450,229-35,711,719 | CN Loss | 261491 | q13.11 - q13.12 | 12.56 | -0.39 | 18 |
| 7065-13 A2 | chr19:35,492,129-35,757,250 | CN Loss | 265122 | q13.11 - q13.12 | 12.39 | -0.34 | 19 |
| 7065-13 A2 | chr19:35,797,281-59,093,239 | LOH | 23295959 | q13.12 - q13.43 | 42.61 | -0.43 | 2079 |
| 12814-15 | chr19:36,044,502-59,093,239 | LOH | 23048738 | q13.12 - q13.43 | 42.87 | -0.30 | 2063 |
| 7065-13 C | chr19:36,044,502-59,093,239 | LOH | 23048738 | q13.12 - q13.43 | 42.87 | -0.41 | 2063 |
| 7065-13 A2 | chr19:36,343,500-37,198,253 | CN Loss | 854754 | q13.12 | 18.79 | -0.48 | 56 |
| 7065-13 C | chr19:36,666,167-37,198,253 | CN Loss | 532087 | q13.12 | 29.49 | -0.58 | 34 |
| 12814-15 | chr19:36,810,479-37,198,253 | CN Loss | 387775 | q13.12 | 18.61 | -0.36 | 26 |
| 7065-13 B | chr19:36,940,760-37,545,614 | CN Loss | 604855 | q13.12 | 19.28 | -0.39 | 40 |
| 12814-15 | chr19:37,213,620-37,545,614 | Homozygous Copy Loss | 331995 | q13.12 | 28.69 | -0.94 | 23 |
| 7065-13 A2 | chr19:37,213,620-37,545,614 | Homozygous Copy Loss | 331995 | q13.12 | 28.69 | -1.45 | 23 |
| 7065-13 C | chr19:37,213,620-37,545,614 | Homozygous Copy Loss | 331995 | q13.12 | 28.69 | -1.35 | 23 |
| 12814-15 | chr19:37,563,211-59,093,239 | CN Loss | 21530029 | q13.12 - q13.43 | 44.47 | -0.29 | 1964 |
| 7065-13 A2 | chr19:37,563,211-59,093,239 | CN Loss | 21530029 | q13.12 - q13.43 | 44.47 | -0.42 | 1964 |
| 7065-13 C | chr19:37,563,211-59,093,239 | CN Loss | 21530029 | q13.12 - q13.43 | 44.47 | -0.41 | 1964 |
| 7065-13 B | chr19:58,741,170-59,093,239 | CN Loss | 352070 | q13.43 | 74.32 | -0.41 | 26 |
| 12814-15 | chr19:6,180,344-10,284,778 | CN Loss | 4104435 | p13.3 - p13.2 | 43.18 | -0.28 | 407 |
| 7065-13 A2 | chr19:6,180,344-10,284,778 | CN Loss | 4104435 | p13.3 - p13.2 | 43.18 | -0.46 | 407 |
| 7065-13 C | chr19:6,180,344-10,284,778 | CN Loss | 4104435 | p13.3 - p13.2 | 43.18 | -0.43 | 407 |
| 12814-15 | chr2:10,195,067-25,580,507 | CN Gain | 15385441 | p25.1 - p23.3 | 24.24 | 0.25 | 1223 |
| 12814-15 | chr2:101,349,854-103,598,575 | CN Loss | 2248722 | q11.2 - q12.1 | 1.49 | -0.15 | 153 |
| 7065-13 B | chr2:103,011,237-105,911,258 | CN Loss | 2900022 | q12.1 | 3.11 | -0.44 | 196 |
| 7065-13 C | chr2:103,011,237-105,911,258 | CN Loss | 2900022 | q12.1 | 3.11 | -0.52 | 196 |
| 7065-13 A2 | chr2:103,211,442-105,911,258 | CN Loss | 2699817 | q12.1 | 3.30 | -0.53 | 181 |
| 12814-15 | chr2:103,619,483-105,911,258 | CN Loss | 2291776 | q12.1 | 3.84 | -0.38 | 156 |
| 7065-13 C | chr2:106,349,806-107,894,008 | CN Gain | 1544203 | q12.2 - q12.3 | 71.04 | 0.23 | 104 |
| 7065-13 C | chr2:113,218,247-120,994,419 | CN Gain | 7776173 | q13 - q14.2 | 21.15 | 0.34 | 566 |
| 12814-15 | chr2:113,232,250-120,968,205 | CN Gain | 7735956 | q13 - q14.2 | 21.18 | 0.27 | 560 |
| 7065-13 A2 | chr2:113,232,250-121,283,724 | CN Gain | 8051475 | q13 - q14.2 | 20.52 | 0.31 | 582 |
| 7065-13 B | chr2:113,232,250-121,283,724 | CN Gain | 8051475 | q13 - q14.2 | 20.52 | 0.28 | 582 |
| 7065-13 A2 | chr2:144,919,995-164,609,151 | LOH | 19689157 | q22.3 - q24.3 | 19.85 | -0.46 | 1386 |
| 7065-13 B | chr2:144,919,995-164,609,151 | LOH | 19689157 | q22.3 - q24.3 | 19.85 | -0.39 | 1386 |
| 12814-15 | chr2:144,919,995-165,074,029 | LOH | 20154035 | q22.3 - q24.3 | 19.49 | -0.35 | 1419 |
| 7065-13 C | chr2:144,919,995-165,074,029 | LOH | 20154035 | q22.3 - q24.3 | 19.49 | -0.48 | 1419 |
| 7065-13 B | chr2:145,299,101-164,488,243 | CN Loss | 19189143 | q22.3 - q24.3 | 20.32 | -0.40 | 1358 |
| 12814-15 | chr2:145,299,101-164,576,841 | CN Loss | 19277741 | q22.3 - q24.3 | 20.23 | -0.37 | 1359 |
| 7065-13 A2 | chr2:145,299,101-164,576,841 | CN Loss | 19277741 | q22.3 - q24.3 | 20.23 | -0.47 | 1359 |
| 7065-13 C | chr2:145,299,101-164,576,841 | CN Loss | 19277741 | q22.3 - q24.3 | 20.23 | -0.49 | 1359 |
| 12814-15 | chr2:208,390,388-209,473,559 | High Copy Gain | 1083172 | q33.3 - q34 | 5.89 | 0.48 | 95 |
| 7065-13 A2 | chr2:209,019,793-220,512,823 | LOH | 11493031 | q34 - q35 | 12.97 | -0.43 | 1079 |
| 7065-13 C | chr2:209,104,230-220,493,861 | LOH | 11389632 | q34 - q35 | 12.92 | -0.46 | 1067 |
| 12814-15 | chr2:209,104,230-220,512,823 | LOH | 11408594 | q34 - q35 | 12.90 | -0.34 | 1070 |
| 7065-13 B | chr2:209,104,230-220,521,602 | LOH | 11417373 | q34 - q35 | 12.89 | -0.36 | 1072 |
| 7065-13 A2 | chr2:209,488,492-210,391,837 | CN Loss | 903346 | q34 | 12.56 | -0.54 | 62 |
| 7065-13 B | chr2:209,488,492-210,421,459 | CN Loss | 932968 | q34 | 12.28 | -0.41 | 65 |
| 7065-13 C | chr2:209,488,492-219,993,736 | CN Loss | 10505245 | q34 - q35 | 12.20 | -0.48 | 995 |
| 12814-15 | chr2:209,488,492-220,228,371 | CN Loss | 10739880 | q34 - q35 | 12.05 | -0.36 | 1009 |
| 7065-13 A2 | chr2:21,494-2,818,117 | CN Gain | 2796624 | p25.3 | 37.46 | 0.14 | 188 |
| 7065-13 A2 | chr2:210,770,094-219,993,736 | CN Loss | 9223643 | q34 - q35 | 12.65 | -0.45 | 909 |
| 7065-13 B | chr2:210,770,094-220,186,656 | CN Loss | 9416563 | q34 - q35 | 12.53 | -0.38 | 920 |
| 12814-15 | chr2:226,130,325-226,631,124 | CN Gain | 500800 | q36.3 | 2.79 | 0.26 | 35 |
| 7065-13 B | chr2:226,163,192-229,291,543 | CN Gain | 3128352 | q36.3 | 15.71 | 0.23 | 241 |
| 7065-13 A2 | chr2:226,349,200-229,254,340 | CN Gain | 2905141 | q36.3 | 16.52 | 0.28 | 228 |
| 7065-13 C | chr2:226,931,562-229,254,340 | CN Gain | 2322779 | q36.3 | 20.15 | 0.35 | 187 |
| 12814-15 | chr2:226,980,376-229,254,340 | CN Gain | 2273965 | q36.3 | 19.90 | 0.24 | 183 |
| 12814-15 | chr2:228,749,278-243,052,331 | LOH | 14303054 | q36.3 - q37.3 | 24.01 | -0.37 | 985 |
| 7065-13 A2 | chr2:228,851,619-243,052,331 | LOH | 14200713 | q36.3 - q37.3 | 23.46 | -0.45 | 978 |
| 7065-13 B | chr2:228,907,543-243,052,331 | LOH | 14144789 | q36.3 - q37.3 | 23.17 | -0.42 | 976 |
| 7065-13 C | chr2:228,922,593-243,052,331 | LOH | 14129739 | q36.3 - q37.3 | 23.19 | -0.50 | 975 |
| 12814-15 | chr2:229,277,943-243,052,331 | CN Loss | 13774389 | q36.3 - q37.3 | 23.75 | -0.39 | 952 |
| 7065-13 A2 | chr2:229,277,943-243,052,331 | CN Loss | 13774389 | q36.3 - q37.3 | 23.75 | -0.46 | 952 |
| 7065-13 C | chr2:229,277,943-243,052,331 | CN Loss | 13774389 | q36.3 - q37.3 | 23.75 | -0.50 | 952 |
| 7065-13 B | chr2:229,310,157-243,052,331 | CN Loss | 13742175 | q36.3 - q37.3 | 23.80 | -0.43 | 950 |
| 12814-15 | chr2:25,597,681-29,449,819 | High Copy Gain | 3852139 | p23.3 - p23.2 | 9.91 | 0.42 | 299 |
| 12814-15 | chr2:82,149,439-85,192,281 | High Copy Gain | 3042843 | p12 - p11.2 | 26.05 | 0.45 | 207 |
| 12814-15 | chr2:95,429,197-105,976,344 | LOH | 10547148 | q11.1 - q12.1 | 33.31 | -0.26 | 886 |
| 7065-13 A2 | chr2:95,429,197-105,976,344 | LOH | 10547148 | q11.1 - q12.1 | 33.31 | -0.36 | 886 |
| 7065-13 B | chr2:95,429,197-105,976,344 | LOH | 10547148 | q11.1 - q12.1 | 33.31 | -0.31 | 886 |
| 7065-13 C | chr2:95,429,197-105,976,344 | LOH | 10547148 | q11.1 - q12.1 | 33.31 | -0.35 | 886 |
| 7065-13 B | chr2:95,429,197-98,562,116 | CN Loss | 3132920 | q11.1 - q11.2 | 83.18 | -0.39 | 162 |
| 7065-13 A2 | chr2:95,429,197-98,575,531 | CN Loss | 3146335 | q11.1 - q11.2 | 83.25 | -0.45 | 163 |
| 7065-13 C | chr2:95,429,197-98,575,531 | CN Loss | 3146335 | q11.1 - q11.2 | 83.25 | -0.45 | 163 |
| 12814-15 | chr2:95,429,197-98,730,815 | CN Loss | 3301619 | q11.1 - q11.2 | 84.04 | -0.31 | 171 |
| 12814-15 | chr2:99,328,763-101,346,696 | CN Loss | 2017934 | q11.2 | 23.98 | -0.39 | 209 |
| 7065-13 B | chr2:99,351,716-101,346,696 | CN Loss | 1994981 | q11.2 | 24.26 | -0.48 | 208 |
| 7065-13 A2 | chr2:99,351,716-101,349,854 | CN Loss | 1998139 | q11.2 | 24.22 | -0.54 | 209 |
| 7065-13 C | chr2:99,365,589-101,370,474 | CN Loss | 2004886 | q11.2 | 24.14 | -0.57 | 209 |
| 12814-15 | chr20:10,194,678-10,504,993 | CN Loss | 310316 | p12.2 | 4.57 | -0.16 | 21 |
| 7065-13 A2 | chr20:20,037,222-20,356,386 | CN Loss | 319165 | p11.23 | 10.01 | -0.24 | 21 |
| 7065-13 B | chr20:24,409,360-24,707,068 | CN Gain | 297709 | p11.21 | 14.55 | 0.23 | 21 |
| 7065-13 B | chr20:31,015,643-62,912,463 | CN Gain | 31896821 | q11.21 - q13.33 | 21.08 | 0.30 | 2383 |
| 12814-15 | chr20:47,039,362-47,384,120 | CN Gain | 344759 | q13.13 | 30.43 | 0.16 | 24 |
| 7065-13 A2 | chr21:14,344,537-48,097,610 | LOH | 33753074 | q11.2 - q22.3 | 28.21 | -0.24 | 2341 |
| 7065-13 A2 | chr21:16,736,936-33,628,270 | CN Loss | 16891335 | q21.1 - q22.11 | 34.47 | -0.22 | 1120 |
| 12814-15 | chr21:19,457,728-19,722,128 | CN Loss | 264401 | q21.1 | 100.00 | -0.18 | 18 |
| 12814-15 | chr21:19,727,879-21,992,124 | CN Gain | 2264246 | q21.1 | 100.00 | 0.14 | 152 |
| 7065-13 B | chr21:22,814,309-23,092,174 | CN Loss | 277866 | q21.1 | 1.24 | -0.24 | 18 |
| 7065-13 C | chr21:28,219,307-29,542,794 | CN Loss | 1323488 | q21.3 | 10.76 | -0.18 | 88 |
| 7065-13 B | chr21:29,043,216-29,338,735 | CN Loss | 295520 | q21.3 | 28.83 | -0.31 | 20 |
| 7065-13 C | chr21:29,564,054-29,821,530 | CN Loss | 257477 | q21.3 | 21.81 | -0.23 | 18 |
| 7065-13 C | chr21:33,048,864-33,374,801 | CN Loss | 325938 | q22.11 | 1.19 | -0.23 | 23 |
| 7065-13 B | chr21:34,001,496-37,320,511 | CN Loss | 3319016 | q22.11 - q22.12 | 9.60 | -0.12 | 277 |
| 7065-13 A2 | chr21:34,001,496-44,346,385 | CN Loss | 10344890 | q22.11 - q22.3 | 10.66 | -0.28 | 817 |
| 7065-13 B | chr21:37,338,220-37,619,724 | CN Loss | 281505 | q22.12 | 67.55 | -0.29 | 19 |
| 7065-13 B | chr21:37,633,823-48,097,610 | CN Loss | 10463788 | q22.12 - q22.3 | 18.04 | -0.12 | 789 |
| 7065-13 C | chr21:40,194,216-40,498,014 | CN Loss | 303799 | q22.2 | 1.71 | -0.25 | 23 |
| 12814-15 | chr21:44,360,107-44,607,103 | CN Gain | 246997 | q22.3 | 14.18 | 0.18 | 18 |
| 7065-13 A2 | chr21:44,710,664-48,097,610 | CN Loss | 3386947 | q22.3 | 36.66 | -0.24 | 249 |
| 7065-13 A2 | chr21:9,648,315-16,435,223 | CN Loss | 6786909 | p11.2 - q21.1 | 33.95 | -0.28 | 101 |
| 12814-15 | chr22:16,054,713-51,213,826 | CN Loss | 35159114 | q11.1 - q13.33 | 41.99 | -0.32 | 2796 |
| 12814-15 | chr22:16,054,713-51,213,826 | LOH | 35159114 | q11.1 - q13.33 | 41.99 | -0.32 | 2796 |
| 7065-13 A2 | chr22:16,054,713-51,213,826 | CN Loss | 35159114 | q11.1 - q13.33 | 41.99 | -0.45 | 2796 |
| 7065-13 A2 | chr22:16,054,713-51,213,826 | LOH | 35159114 | q11.1 - q13.33 | 41.99 | -0.45 | 2796 |
| 7065-13 B | chr22:16,054,713-51,213,826 | CN Loss | 35159114 | q11.1 - q13.33 | 41.99 | -0.39 | 2796 |
| 7065-13 B | chr22:16,054,713-51,213,826 | LOH | 35159114 | q11.1 - q13.33 | 41.99 | -0.39 | 2796 |
| 7065-13 C | chr22:16,054,713-51,213,826 | CN Loss | 35159114 | q11.1 - q13.33 | 41.99 | -0.45 | 2796 |
| 7065-13 C | chr22:16,054,713-51,213,826 | LOH | 35159114 | q11.1 - q13.33 | 41.99 | -0.45 | 2796 |
| 7065-13 B | chr3:113,010,466-113,288,430 | CN Loss | 277965 | q13.2 | 1.13 | -0.24 | 20 |
| 7065-13 B | chr3:116,309,057-116,999,611 | CN Loss | 690555 | q13.31 | 3.69 | -0.45 | 47 |
| 12814-15 | chr3:140,476,691-197,852,564 | LOH | 57375874 | q23 - q29 | 19.40 | -0.38 | 4320 |
| 7065-13 A2 | chr3:140,505,491-197,852,564 | LOH | 57347074 | q23 - q29 | 19.36 | -0.50 | 4318 |
| 7065-13 C | chr3:140,505,491-197,852,564 | LOH | 57347074 | q23 - q29 | 19.36 | -0.51 | 4318 |
| 7065-13 B | chr3:140,806,967-197,852,564 | LOH | 57045598 | q23 - q29 | 19.34 | -0.42 | 4299 |
| 7065-13 B | chr3:140,941,407-162,425,249 | CN Loss | 21483843 | q23 - q26.1 | 14.98 | -0.43 | 1505 |
| 7065-13 A2 | chr3:141,049,321-162,418,405 | CN Loss | 21369085 | q23 - q26.1 | 15.02 | -0.51 | 1497 |
| 12814-15 | chr3:141,049,321-197,852,564 | CN Loss | 56803244 | q23 - q29 | 19.43 | -0.38 | 4283 |
| 7065-13 C | chr3:141,049,321-197,852,564 | CN Loss | 56803244 | q23 - q29 | 19.43 | -0.51 | 4283 |
| 7065-13 A2 | chr3:162,425,249-162,702,814 | Homozygous Copy Loss | 277566 | q26.1 | 100.00 | -0.93 | 19 |
| 7065-13 B | chr3:162,440,045-162,702,814 | Homozygous Copy Loss | 262770 | q26.1 | 100.00 | -0.84 | 18 |
| 7065-13 D | chr3:162,440,045-162,702,814 | CN Loss | 262770 | q26.1 | 100.00 | -0.44 | 18 |
| 7065-13 A2 | chr3:162,719,684-197,852,564 | CN Loss | 35132881 | q26.1 - q29 | 21.41 | -0.50 | 2767 |
| 7065-13 B | chr3:162,719,684-197,852,564 | CN Loss | 35132881 | q26.1 - q29 | 21.41 | -0.42 | 2767 |
| 12814-15 | chr3:17,944,858-18,233,716 | CN Gain | 288859 | p24.3 | 0.31 | 0.14 | 20 |
| 7065-13 B | chr3:32,344,306-32,618,146 | CN Loss | 273841 | p22.3 | 1.78 | -0.22 | 21 |
| 7065-13 B | chr3:47,262,246-47,598,663 | CN Loss | 336418 | p21.31 | 100.00 | -0.23 | 24 |
| 7065-13 B | chr3:56,459,364-56,692,321 | CN Loss | 232958 | p14.3 | 14.04 | -0.23 | 18 |
| 7065-13 B | chr3:62,184,407-77,165,296 | CN Gain | 14980890 | p14.2 - p12.3 | 17.59 | 0.28 | 1372 |
| 7065-13 C | chr3:62,369,495-69,932,226 | CN Gain | 7562732 | p14.2 - p13 | 12.30 | 0.30 | 739 |
| 7065-13 A2 | chr3:62,369,495-77,165,296 | CN Gain | 14795802 | p14.2 - p12.3 | 17.81 | 0.29 | 1360 |
| 12814-15 | chr3:62,661,513-77,165,296 | CN Gain | 14503784 | p14.2 - p12.3 | 18.15 | 0.25 | 1340 |
| 7065-13 C | chr3:69,948,677-77,165,296 | CN Gain | 7216620 | p13 - p12.3 | 23.62 | 0.33 | 596 |
| 7065-13 A2 | chr3:77,764,672-86,533,198 | LOH | 8768527 | p12.3 - p12.1 | 11.28 | -0.41 | 642 |
| 7065-13 C | chr3:78,156,918-86,533,198 | LOH | 8376281 | p12.3 - p12.1 | 11.37 | -0.42 | 616 |
| 12814-15 | chr3:78,167,327-86,508,755 | LOH | 8341429 | p12.3 - p12.1 | 11.42 | -0.30 | 614 |
| 7065-13 B | chr3:78,167,327-86,508,755 | LOH | 8341429 | p12.3 - p12.1 | 11.42 | -0.32 | 614 |
| 7065-13 A2 | chr3:78,421,880-81,642,131 | CN Loss | 3220252 | p12.3 - p12.2 | 1.86 | -0.48 | 274 |
| 7065-13 C | chr3:78,421,880-81,642,131 | CN Loss | 3220252 | p12.3 - p12.2 | 1.86 | -0.48 | 274 |
| 7065-13 B | chr3:78,546,548-81,642,131 | CN Loss | 3095584 | p12.3 - p12.2 | 1.37 | -0.36 | 265 |
| 12814-15 | chr3:78,620,239-81,642,131 | CN Loss | 3021893 | p12.3 - p12.2 | 1.29 | -0.36 | 261 |
| 12814-15 | chr3:81,650,488-82,237,438 | CN Gain | 586951 | p12.2 | 1.65 | 0.22 | 40 |
| 7065-13 B | chr3:81,650,488-82,237,438 | CN Gain | 586951 | p12.2 | 1.65 | 0.19 | 40 |
| 7065-13 C | chr3:81,650,488-82,312,436 | CN Gain | 661949 | p12.2 | 1.46 | 0.15 | 45 |
| 12814-15 | chr3:82,257,693-85,776,919 | CN Loss | 3519227 | p12.2 - p12.1 | 24.84 | -0.35 | 236 |
| 7065-13 B | chr3:82,257,693-85,776,919 | CN Loss | 3519227 | p12.2 - p12.1 | 24.84 | -0.40 | 236 |
| 7065-13 A2 | chr3:82,330,233-85,729,502 | CN Loss | 3399270 | p12.2 - p12.1 | 25.72 | -0.48 | 228 |
| 7065-13 C | chr3:82,330,233-85,729,502 | CN Loss | 3399270 | p12.2 - p12.1 | 25.72 | -0.50 | 228 |
| 7065-13 C | chr3:85,753,018-86,345,901 | CN Gain | 592884 | p12.1 | 0.83 | 0.33 | 40 |
| 7065-13 A2 | chr3:85,753,018-86,367,480 | CN Gain | 614463 | p12.1 | 0.80 | 0.30 | 41 |
| 12814-15 | chr3:85,787,399-87,691,945 | High Copy Gain | 1904547 | p12.1 - p11.2 | 2.19 | 0.44 | 127 |
| 7065-13 B | chr3:85,787,399-87,691,945 | High Copy Gain | 1904547 | p12.1 - p11.2 | 2.19 | 0.47 | 127 |
| 7065-13 C | chr3:86,367,480-87,064,666 | High Copy Gain | 697187 | p12.1 | 2.82 | 0.87 | 48 |
| 7065-13 A2 | chr3:86,376,976-87,064,666 | High Copy Gain | 687691 | p12.1 | 2.86 | 0.83 | 47 |
| 7065-13 A2 | chr3:87,079,214-87,713,347 | CN Gain | 634134 | p12.1 - p11.2 | 3.18 | 0.33 | 43 |
| 7065-13 C | chr3:87,079,214-87,713,347 | CN Gain | 634134 | p12.1 - p11.2 | 3.18 | 0.40 | 43 |
| 7065-13 B | chr3:87,713,347-88,758,307 | CN Loss | 1044961 | p11.2 - p11.1 | 7.48 | -0.31 | 71 |
| 12814-15 | chr3:87,713,347-88,774,790 | CN Loss | 1061444 | p11.2 - p11.1 | 7.37 | -0.27 | 72 |
| 7065-13 A2 | chr3:87,731,430-88,758,307 | CN Loss | 1026878 | p11.2 - p11.1 | 7.61 | -0.47 | 70 |
| 7065-13 C | chr3:87,731,430-88,758,307 | CN Loss | 1026878 | p11.2 - p11.1 | 7.61 | -0.46 | 70 |
| 7065-13 A2 | chr3:88,774,790-90,473,621 | CN Gain | 1698832 | p11.1 | 42.91 | 0.34 | 153 |
| 7065-13 B | chr3:88,774,790-90,473,621 | CN Gain | 1698832 | p11.1 | 42.91 | 0.28 | 153 |
| 7065-13 C | chr3:88,774,790-90,473,621 | CN Gain | 1698832 | p11.1 | 42.91 | 0.37 | 153 |
| 12814-15 | chr3:88,793,982-90,473,621 | CN Gain | 1679640 | p11.1 | 43.40 | 0.28 | 152 |
| 7065-13 C | chr3:93,517,443-100,462,575 | CN Loss | 6945133 | q11.1 - q12.2 | 16.76 | -0.44 | 471 |
| 7065-13 C | chr3:93,517,443-100,526,582 | LOH | 7009140 | q11.1 - q12.2 | 17.16 | -0.44 | 480 |
| 7065-13 C | chr4:109,732,656-112,814,763 | CN Loss | 3082108 | q25 | 8.03 | -0.32 | 211 |
| 7065-13 A2 | chr4:134,920,993-135,230,285 | CN Loss | 309293 | q28.3 | 88.81 | -0.68 | 22 |
| 7065-13 C | chr4:134,920,993-135,230,285 | CN Loss | 309293 | q28.3 | 88.81 | -0.66 | 22 |
| 7065-13 D | chr4:134,931,779-135,230,285 | CN Loss | 298507 | q28.3 | 88.41 | -0.82 | 21 |
| 7065-13 C | chr4:31,700,094-31,966,506 | CN Gain | 266413 | p15.1 | 4.72 | 0.20 | 18 |
| 7065-13 B | chr4:45,430,867-45,670,531 | CN Gain | 239665 | p12 | 3.02 | 0.17 | 18 |
| 7065-13 B | chr4:56,231,119-56,512,857 | CN Loss | 281739 | q12 | 8.35 | -0.21 | 19 |
| 7065-13 A2 | chr5:1,820,443-2,376,332 | CN Gain | 555890 | p15.33 | 19.28 | 0.18 | 40 |
| 7065-13 C | chr5:135,611,442-180,698,312 | LOH | 45086871 | q31.1 - q35.3 | 19.03 | -0.45 | 3285 |
| 7065-13 C | chr5:135,761,681-180,698,312 | CN Loss | 44936632 | q31.1 - q35.3 | 19.09 | -0.45 | 3275 |
| 12814-15 | chr5:157,727,648-158,044,628 | CN Loss | 316981 | q33.3 | 0.08 | -0.22 | 23 |
| 7065-13 B | chr5:37,427,143-37,713,302 | CN Loss | 286160 | p13.2 | 23.11 | -0.22 | 18 |
| 7065-13 C | chr6:143,721,486-143,981,926 | CN Gain | 260441 | q24.2 | 13.96 | 0.12 | 18 |
| 12814-15 | chr6:34,100,568-45,545,975 | LOH | 11445408 | p21.31 - p21.1 | 12.48 | -0.38 | 904 |
| 7065-13 B | chr6:34,129,776-45,530,471 | LOH | 11400696 | p21.31 - p21.1 | 12.53 | -0.43 | 902 |
| 7065-13 A2 | chr6:34,129,776-45,777,475 | LOH | 11647700 | p21.31 - p21.1 | 12.28 | -0.47 | 918 |
| 7065-13 C | chr6:34,151,564-45,653,263 | LOH | 11501700 | p21.31 - p21.1 | 12.42 | -0.50 | 909 |
| 12814-15 | chr6:34,332,179-45,265,960 | CN Loss | 10933782 | p21.31 - p21.1 | 12.43 | -0.39 | 858 |
| 7065-13 B | chr6:34,332,179-45,273,877 | CN Loss | 10941699 | p21.31 - p21.1 | 12.42 | -0.45 | 859 |
| 7065-13 A2 | chr6:34,343,566-45,273,877 | CN Loss | 10930312 | p21.31 - p21.1 | 12.42 | -0.50 | 858 |
| 7065-13 C | chr6:34,351,767-45,273,877 | CN Loss | 10922111 | p21.31 - p21.1 | 12.43 | -0.52 | 857 |
| 7065-13 B | chr6:94,112,979-94,332,620 | CN Loss | 219642 | q16.1 | 100.00 | -0.22 | 19 |
| 7065-13 B | chr7:154,030,823-159,118,443 | CN Gain | 5087621 | q36.2 - q36.3 | 31.61 | 0.12 | 353 |
| 7065-13 A2 | chr7:2,227,707-2,506,595 | CN Gain | 278889 | p22.3 | 3.97 | 0.24 | 19 |
| 7065-13 A2 | chr7:86,608,561-86,843,211 | CN Loss | 234651 | q21.12 | 0.42 | -0.26 | 20 |
| 7065-13 B | chr8:96,704,278-96,983,076 | CN Loss | 278799 | q22.1 | 5.40 | -0.19 | 19 |
| 7065-13 C | chr9:10,044,092-20,322,257 | CN Loss | 10278166 | p23 - p21.3 | 24.87 | -0.50 | 769 |
| 7065-13 A2 | chr9:111,070,724-111,368,482 | CN Loss | 297759 | q31.2 - q31.3 | 1.25 | -0.25 | 23 |
| 12814-15 | chr9:123,879,841-124,108,959 | CN Loss | 229119 | q33.2 | 0.01 | -0.27 | 25 |
| 7065-13 C | chr9:139,830,940-140,092,057 | CN Loss | 261118 | q34.3 | 100.00 | -0.31 | 18 |
| 7065-13 B | chr9:139,867,698-140,136,965 | CN Loss | 269268 | q34.3 | 100.00 | -0.26 | 32 |
| 7065-13 A2 | chr9:139,867,698-140,151,044 | CN Loss | 283347 | q34.3 | 100.00 | -0.26 | 37 |
| 7065-13 C | chr9:140,839,842-141,054,761 | CN Gain | 214920 | q34.3 | 81.61 | 0.18 | 35 |
| 7065-13 C | chr9:20,338,429-21,255,150 | Homozygous Copy Loss | 916722 | p21.3 | 65.30 | -1.23 | 104 |
| 12814-15 | chr9:204,738-21,700,795 | CN Loss | 21496058 | p24.3 - p21.3 | 33.47 | -0.36 | 1849 |
| 12814-15 | chr9:204,738-34,290,028 | LOH | 34085291 | p24.3 - p13.3 | 38.01 | -0.38 | 2879 |
| 7065-13 B | chr9:204,738-36,655,648 | CN Loss | 36450911 | p24.3 - p13.2 | 36.20 | -0.27 | 3047 |
| 7065-13 B | chr9:204,738-36,855,486 | LOH | 36650749 | p24.3 - p13.2 | 36.00 | -0.27 | 3063 |
| 7065-13 A2 | chr9:204,738-39,184,065 | LOH | 38979328 | p24.3 - p13.1 | 36.64 | -0.46 | 3225 |
| 7065-13 C | chr9:204,738-39,184,065 | LOH | 38979328 | p24.3 - p13.1 | 36.64 | -0.53 | 3225 |
| 7065-13 A2 | chr9:204,738-44,893,094 | CN Loss | 44688357 | p24.3 - p11.2 | 44.73 | -0.46 | 3229 |
| 7065-13 C | chr9:204,738-9,749,627 | CN Loss | 9544890 | p24.3 - p23 | 35.91 | -0.49 | 926 |
| 7065-13 C | chr9:21,265,500-21,700,795 | CN Loss | 435296 | p21.3 | 100.00 | -0.47 | 31 |
| 12814-15 | chr9:21,712,193-22,054,015 | Homozygous Copy Loss | 341823 | p21.3 | 100.00 | -1.00 | 167 |
| 7065-13 C | chr9:21,712,193-23,371,941 | Homozygous Copy Loss | 1659749 | p21.3 | 50.12 | -1.17 | 256 |
| 12814-15 | chr9:22,056,019-33,061,716 | CN Loss | 11005698 | p21.3 - p21.1 | 44.34 | -0.37 | 784 |
| 7065-13 C | chr9:23,386,738-44,893,094 | CN Loss | 21506357 | p21.3 - p11.2 | 55.51 | -0.50 | 1124 |
| 7065-13 C | chr9:9,763,352-10,025,975 | Homozygous Copy Loss | 262624 | p23 | 63.73 | -1.09 | 19 |
| 7065-13 B | chr9:92,682,813-92,961,558 | CN Loss | 278746 | q22.2 | 0.38 | -0.20 | 19 |
| 7065-13 B | chrX:110,491,152-146,504,200 | CN Loss | 36013049 | q23 - q27.3 | 26.29 | -0.22 | 2573 |
| 7065-13 B | chrX:151,061,540-151,304,944 | CN Loss | 243405 | q28 | 51.46 | -0.29 | 32 |
| 7065-13 B | chrX:177,942-1,597,685 | CN Gain | 1419744 | p22.33 | 42.39 | 0.21 | 50 |
| 7065-13 C | chrX:177,942-1,597,685 | CN Gain | 1419744 | p22.33 | 42.39 | 0.26 | 50 |
| 7065-13 B | chrX:61,732,394-146,611,545 | LOH | 84879152 | q11.1 - q27.3 | 23.85 | -0.24 | 6329 |
| 7065-13 B | chrX:61,732,394-62,474,000 | CN Loss | 741607 | q11.1 | 65.59 | -0.42 | 39 |
| 12814-15 | chrX:61,732,394-67,285,971 | LOH | 5553578 | q11.1 - q12 | 24.42 | -0.02 | 634 |
| 7065-13 D | chrX:61,732,394-67,300,619 | LOH | 5568226 | q11.1 - q12 | 24.43 | -0.02 | 635 |
| 7065-13 C | chrX:61,732,394-67,331,775 | LOH | 5599382 | q11.1 - q12 | 24.85 | -0.04 | 637 |
| 7065-13 A2 | chrX:61,732,394-67,423,321 | LOH | 5690928 | q11.1 - q12 | 25.72 | -0.08 | 644 |
| 7065-13 B | chrX:62,885,779-63,566,929 | CN Loss | 681151 | q11.1 - q11.2 | 1.02 | -0.33 | 62 |
| 7065-13 B | chrX:64,806,769-110,398,019 | CN Loss | 45591251 | q12 - q23 | 22.29 | -0.25 | 3510 |
| 7065-13 B | chrX:9,559,263-9,761,062 | CN Gain | 201800 | p22.2 | 6.45 | 0.13 | 18 |
